# Skull Base Tumors and Tumor-Like Lesions: A Pictorial Review

**DOI:** 10.12659/PJR.901937

**Published:** 2017-07-25

**Authors:** Akira Kunimatsu, Natsuko Kunimatsu

**Affiliations:** 1 Department of Radiology, Graduate School of Medicine, The University of Tokyo, Tokyo, Japan; 2 Department of Radiology, Mita Hospital, International University of Health and Welfare, Tokyo, Japan

**Keywords:** Magnetic Resonance Imaging, Skull Base Neoplasms, Tomography, Spiral Computed

## Abstract

A number of tumors and tumor-like non-neoplastic lesions with different cell types on histology occur in the skull base. A wide variety in disease and lesion appearance often complicates the process of radiological diagnosis. The main role of radiographic imaging is the detection and characterization of skull base lesions, with evaluation of the extent of invasion or preservation of adjacent critical organs. Evaluation of the skull base anatomy and surgical planning by using image guidance are also important for surgeons. Computed tomography (CT) and magnetic resonance (MR) imaging are the preferred modalities for the evaluation of skull base lesions. CT and MR are used for lesion detection, tissue characterization and assessment of neurovascular and bone involvement by the lesions. Both modalities provide useful information, one sometimes of greater value than the other. T1-weighted MR imaging is useful in detecting skull base lesions, typically surrounded by abundant fatty bone marrow. T2-weighted MR imaging is generally useful for tumor tissue characterization. CT surpasses MR imaging in evaluating intratumoral calcification and bone destruction or hyperostosis. To date, imaging features have been well-reported in individual skull base tumors; however, correct diagnosis by imaging alone still presents a challenge. Knowledge of clinical issues and awareness of variants of skull base tumors are of help in making a diagnosis. The purpose of this article is to review pertinent clinical issues, typical imaging appearances and certain imaging variations of common skull base lesions.

## Background

The skull base is the anatomic junction of the neural and facial viscerocranium. It has clinically unique importance because it supports the brain and contains the neurovascular structures entering or exiting the skull.

Skull base lesions may originate within the skull base or involve it by growth from either the intracranial dura or extracranial structures. A number of tumors and tumor-like non-neoplastic lesions, with different cell types, can thus affect the skull base. This wide variety of lesions often confuses beginners and experts alike. The purpose of imaging includes detection, localization and characterization of skull base lesions. Estimation of tumor extent and invasion to critical organs is important as well. These goals are achievable through the use of computed tomography (CT) and magnetic resonance (MR) imaging; one complements the other. T1-weighted MR imaging is useful in detecting skull base lesions typically surrounded by abundant fatty bone marrow. Solid tumors with marked hyperintensity on T2-weighted images are likely to contain chondroid, chordoid or myxoid matrix, while hypointensity suggests abundant fibrous tissue or hemosiderin within the tumor. CT surpasses MR imaging in evaluating intratumoral calcification and bone destruction or osteosclerosis. Dural-based tumors with hyperostosis of the underlying bones very often suggest meningiomas. Even now, when imaging plays an important role in the diagnosis process, knowledge and awareness of clinical issues and variants of skull base tumors are also of help. The first purpose of this article is to discuss the usefulness of non-contrast T1-weighted MR imaging in detecting skull base lesions and the second is to review pertinent clinical issues, typical imaging appearances and certain imaging variants regarding skull base tumors and tumor-like lesions.

## The Rationale for Imaging

Skull base is generally examined using CT, MR imaging or both. Other than diagnosis by using each modality, multimodal image fusion techniques across CT, MR imaging and, in some cases, conventional angiography, are recently favored by neurosurgeons as an intraoperative image guidance [1]. ^18^F-fluoro-deoxyglucose positron emission tomography (PET) or PET-CT is often performed to assess metabolic activity of skull base lesions and to locate the primary tumor or other similar lesions located in areas other than the skull base. The purpose of CT and MR imaging is to evaluate *(a)* lesion characterization, *(b)* paranasal sinus involvement, *(c)* involvement of extracranial soft tissues, *(d)* bone involvement, *(e)* neurovascular involvement, *(f)* invasion to brain and *(g)* variation of skull base anatomy [2].

## Normal Bone Marrow Development and Variation of The Skull Base

The skull base is comprised of the frontal, ethmoid, sphenoid, temporal and occipital bones. The frontal and ethmoid bones compose the anterior skull base. The middle skull base is formed by the sphenoid bone and the anterior aspect of the temporal bone. The occipital bone and the posterior aspect of the temporal bone constitute the posterior skull base.

**Figure 1:**
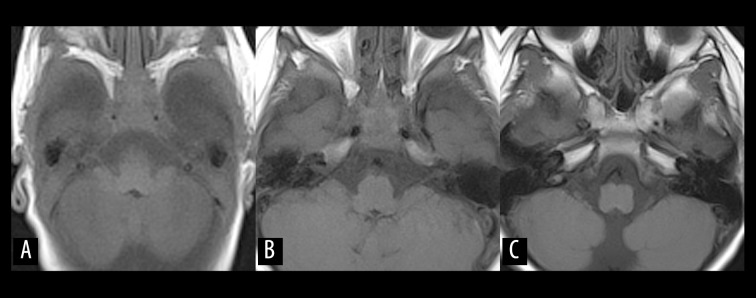
Normal fatty bone marrow infiltration of the skull base in childhood. (**A–C**) Non-contrast, T1-weighted images of a 3-month-old baby (**A**), and 2.5-year-old (**B**) and 5-year-old (**C**) girls demonstrate serial signal change from iso- to hyperintensity of the bone marrow at the skull base, corresponding to normal conversion from cellular to fatty marrow in childhood.

**Figure 2:**
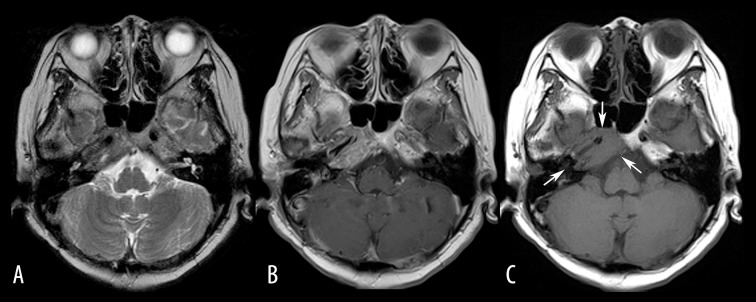
Bone metastasis to the skull base in a 53-year-old woman with a history of breast cancer. (**A, B**) On axial T2-weighted (**A**) and contrast-enhanced T1-weighted (**B**) MR images, MR signals from the metastatic bone tumor of the right petrous apex resemble those from the bone marrow in the contralateral normal petrous apex. (**C**) Non-contrast, T1-weighted (**C**) MR image most clearly delineates the hypointense tumor (arrows).

It is widely known that conversion from cellular to fatty bone marrow begins early after birth at the clivus and the calvaria [3]. The clivus, one of the key components of the skull base, is comprised of the basisphenoid and the basioccipital bones and contains abundant medullary cavity. Non-contrast T1-weighted MR imaging is very sensitive to bone marrow conversion in childhood (Figure 1). An early MR study on cranial bone marrow in children has shown that the marrow with uniform cellular marrow signal is no longer observed in the clivus or the calvaria after the age of seven years [3]. Most skull base tumors show intermediate to low signals on non-contrast T1-weighted MR images and therefore skull base lesions are often very clearly delineated by surrounding hyperintense fatty marrow (Figure 2).

The process of paranasal sinus, mastoid and accessory skull base pneumatization follows marrow conversion, developing from infancy to adolescence. Accessory pneumatization and absent or hypoplastic sinuses are developmental variants that are occasionally seen. Arrested pneumatization is another less recognized variation that may simulate disease [4]; it corresponds to failure of pneumatization before respiratory mucosa has fully extended into developing sinuses. Arrested pneumatization typically appears as a non-expansile lesion with osteosclerotic borders, internal fat and curvilinear calcifications in the basisphenoid bone or adjacent skull base [4].

## The Basics of Skull Base Lesions

In the following section, basic clinical knowledge and imaging features of skull base lesions will be reviewed based on typical locations where the lesions occur. Otolaryngologic lesions that typically occur in the temporal bone are not included here for the sake of conciseness.

## Anterior Skull Base

### Olfactory neuroblastoma

Olfactory neuroblastoma or esthesioneuroblastoma is a malignant neoplasm of the neural crest origin arising from the olfactory epithelium located in the upper nasal fossa [5]. It accounts for 3–5% of all intranasal neoplasms [6,7]. It has bimodal incidence peaks in the teens and 40s with a slight female predominance [6]. The typical clinical presentation is nasal stuffiness (70%) and bleeding (46%) [7]. Because of the high local recurrence rate, treatment includes surgery and radiation therapy even for a small tumor confined to the nasal cavity [7].

**Figure 3:**
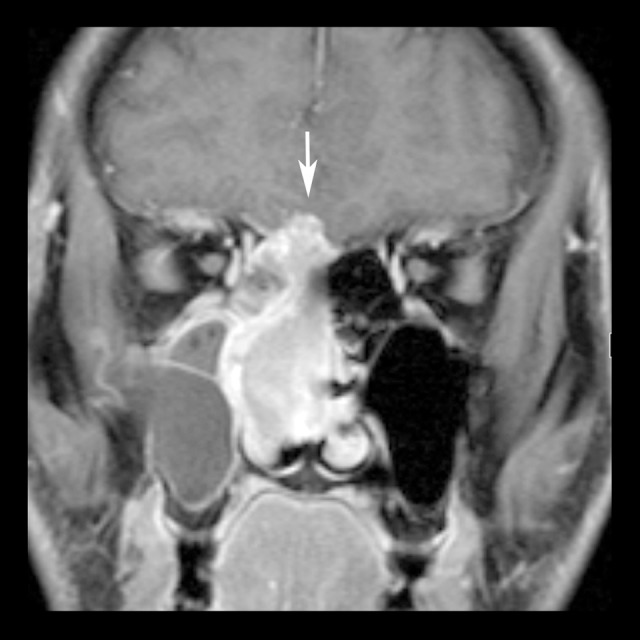
Olfactory neuroblastoma in a 38-year-old man. Coronal post-contrast, fat-suppressed, T1-weighted image shows homogenously enhancing tumor in the right nasal cavity with intracranial extension (arrow).

Olfactory neuroblastoma appears as iso- to slightly hyperdense on non-contrast CT, iso- to hypointense on non-contrast T1-weighted MR images and hyper- to isointense on T2-weighted MR images (Figure 3). Intratumoral cyst formation or necrosis is not uncommon [8]. Intracranial extension of olfactory neuroblastoma sometimes has characteristic marginal cysts where the tumor approaches the brain [9]. On contrast-enhanced T1-weighted images, the tumor shows homogeneous enhancement but can be inhomogeneous with necrosis. Diffusion-weighted imaging typically shows restricted diffusion.

## Anterior and Middle Skull Base

### Skull base invasion from head and neck tumor

Head and neck tumors sometimes invade the anterior or the middle skull base by either direct invasion or perineural tumor spread. Perineural tumor spread into the intracranial space can occur in adenoid cystic carcinoma, squamous cell carcinoma, lymphoma and melanoma [10]. It is most common in patients aged 40 to 60 years with a male predominance and typically spreads along the branches of the trigeminal nerve or the facial nerve.

**Figure 4:**
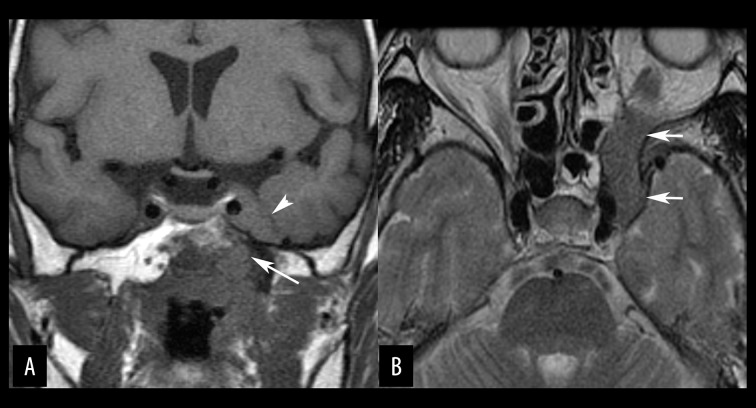
Perineural tumor spread of nasopharyngeal carcinoma in a 63-year-old woman. (**A**) Coronal, non-contrast, T1-weighted, MR image shows nasopharyngeal mucosal wall thickening. The tumor invades into the basisphenoid and spreads through the left Vidian canal (arrow) to the pterygopalatine fossa (not shown), and then goes up to the left cavernous sinus along the maxillary branch of the left trigeminal nerve. The left cavernous sinus is also replaced by the tumor (arrow head). (**B**) Axial, T2-weighted MR image shows the mass with intermediate intensity along the maxillary branch of the left trigeminal nerve (arrows).

CT typically shows the expansion of the skull base foramen with an enlarged nerve in it. Non-contrast T1-weighted MR imaging demonstrates a hypointense, enlarged nerve with a loss of perineural fat. The enlarged nerve shows variable intensity on T2-weighted MR imaging. A tubular, enhanced mass is shown on contrast-enhanced MR imaging (Figure 4) [11].

### Fibrous dysplasia

Fibrous dysplasia (FD) can affect any bones in the body; the skull and the facial bones are affected in 10–25% of patients with monostotic FD and in 50% of patients with polyostotic FD [12]. FD most commonly presents in the teens or 20s with female predominance but can be incidentally found in the elderly on CT or MR imaging performed for other purposes. Symptoms depend on the site of the lesion. Observation is chosen in asymptomatic patients. Curettage or volume reduction surgery may be employed if there is bone pain, skeletal deformity or progressive neurological deficit.

**Figure 5:**
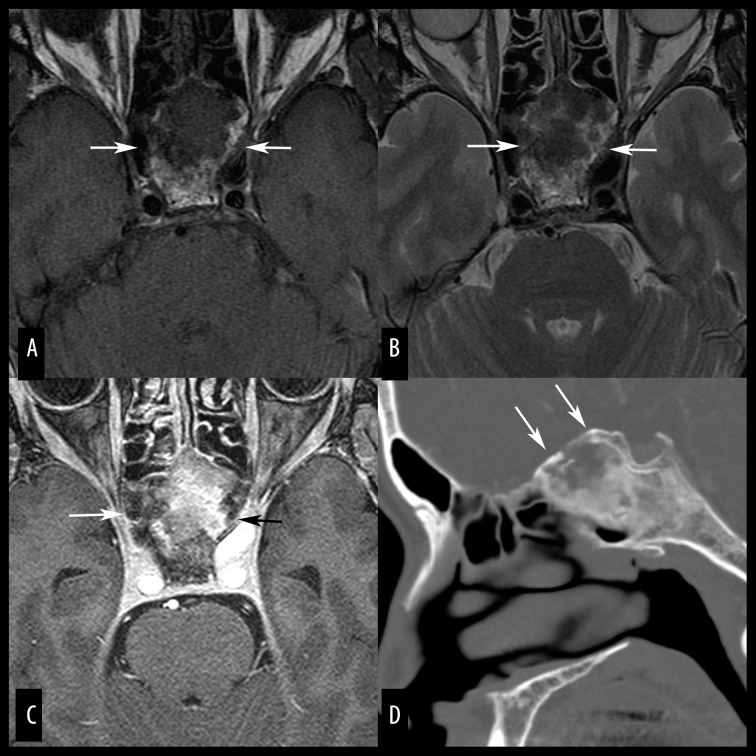
Fibrous dysplasia of the sphenoid in a 61-year-old man. (**A, B**) Axial, T1-weighted (**A**) and T2-weighted (**B**) MR images show a mass lesion (arrows) of the sphenoid with mixed intensity ranging from hypo- to hyperintense. (**C**) Axial, fat-suppressed, contrast-enhanced MR image reveals inhomogeneous but strong enhancement of the mass (arrows). (**D**) Sagittal-reformatted bone window CT image shows a mixed ground-glass and cystic appearance that is typical of fibrous dysplasia. A convex margin (arrows) also suggests fibrous dysplasia rather than arrested pneumatization. Fibrous dysplasia incidentally found in the elderly can be mistaken for a neoplasm on MR images.

FD shows three dominant patterns on CT: the ground-glass pattern (56%), the homogeneously sclerotic pattern (23%) and the predominantly cystic pattern (21%) [12]. FD often appears as a mixture of these patterns and may show variable appearances on CT. FD is predominantly hypointense on both non-contrast T1-weighted and T2-weighted MR images. Non-mineralized areas and regions of cystic changes show hyperintensity on T2-weighted MR images. Heterogeneous enhancement is sometimes seen on contrast-enhanced T1-weighted MR images, which can be easily confused with a skull base neoplasm (Figure 5) [12].

## Middle Skull Base

### Pituitary adenoma

**Figure 6:**
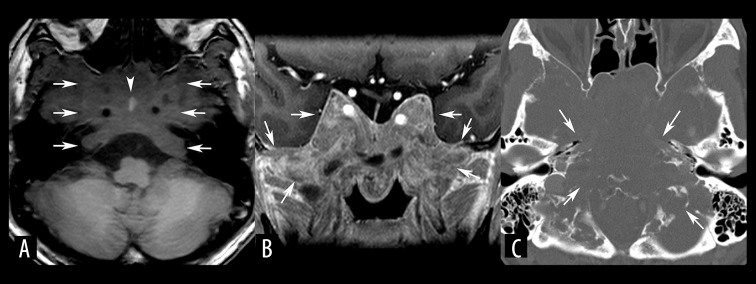
Pituitary adenoma in a 38-year-old man. (**A**) Axial, non-contrast, T1-weighted MR image shows a large, infiltrating, hypointense mass (arrows) at the central skull base. Bone invasion can be confirmed by the loss of normal high signals from fatty bone marrow. Intratumoral hemorrhage (arrow head) can be also seen. (**B**) On coronal-reformatted, contrast-enhanced MR image, the mass shows moderate enhancement with a very indistinct margin (arrows). (**C**) Axial, bone window CT image shows bone destruction of the central skull base (arrows). A pathological analysis revealed a prolactin-producing pituitary adenoma.

Pituitary adenomas are benign tumors arising from the anterior lobe of the pituitary gland. They are the most common cause of sellar masses that occur from the age of 20 years, accounting for up to 10% of all intracranial neoplasms [13]. Intrasellar or combined intra- and suprasellar location is the most common form of pituitary adenomas. A subgroup of giant pituitary adenomas, called invasive pituitary adenoma, may show aggressive invasion into the skull base and thus can be easily mistaken for other skull base malignancies (Figure 6) [14,15].

Clinical symptoms are caused by the local mass effects and/or hormonal abnormalities. Prolactin-producing adenoma accounts for 57% of all pituitary adenomas [16]. A dopamine agonist should be used for initial treatment for prolactin-producing pituitary adenomas [17]. Trans-sphenoidal surgery is most commonly employed for initial treatment of other pituitary adenomas. Radiation therapy is employed when a residual tumor grows progressively.

Pituitary adenomas typically show iso- to mild hyperdensity on non-contrast CT, iso- to hypointensity on T1-weighted MR images and mild hyper- to isointensity on T2-weighted MR images. They sometimes contain intratumoral cysts or hemorrhage. Contrast enhancement of the tumor is moderate and mostly homogeneous. Large adenomas expand the sella. Invasive pituitary adenomas may erode or destroy adjacent bones.

### Chondrosarcoma

**Figure 7:**
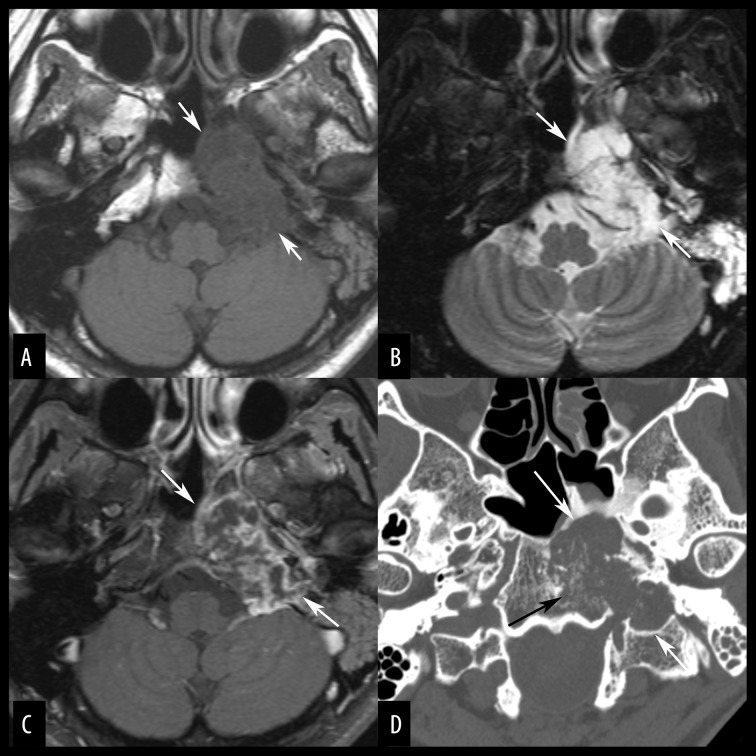
Skull base chondrosarcoma in a 46-year-old woman. (**A**) Axial, non-contrast, T1-weighted MR image shows an expansile, hypointense mass (arrows) engulfing the left petrous apex and the clivus. The epicenter of the tumor is likely located at the left petroclival fissure. (**B**) On an axial, fat-suppressed, T2-weighted MR image, most of the tumor shows very high signals (arrows). The left mastoid air cells lose aeration, likely because the Eustachian tube is obstructed. (**C**) Axial, contrast-enhanced, MR image with fat suppression shows peripheral enhancement (arrows). (**D**) On an axial, bone window CT image, punctuate calcification is seen in the tumor (black arrow). The margin of the tumor is rather distinct (white arrows).

**Figure 8:**
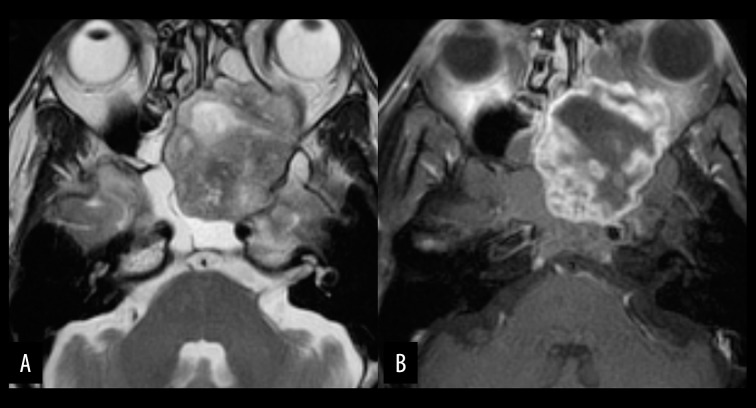
Chondrosarcoma of the sphenoid in a 56-year-old man. (**A**) Axial, T2-weighted MR image shows a lobulated mass at the sphenoid (arrows). The mass contains abundant intermediate signals but also includes hyperintense foci. (**B**) Axial, contrast-enhanced, MR image with fat suppression shows peripheral enhancement that indicates chondrogenic tumor. A pathological analysis revealed grade 1 chondrosarcoma.

Skull base chondrosarcoma is thought to arise from the cartilage, bones with endochondral ossification and primitive mesenchymal cells of the meninges. It accounts for 6% of skull base tumors [18] and can occur at all ages from the teens into the 90s. No sex predominance has been reported. Skull base chondrosarcoma most commonly occurs along the petrooccipital fissure (Figure 7). The sphenoid is the next most common location (Figure 8) but is far rarer [18]. Typical clinical symptoms include abducens nerve palsy and headache [18]. Surgery and adjuvant radiation therapy are most commonly employed for treatment [18]. Skull base chondrosarcoma is well-differentiated, grade 1 chondrosarcoma in many cases, and the prognosis is rather favorable [18].

Skull base chondrosarcoma typically forms an expansile mass with multilobulated margins. CT shows an osteolytic mass. Typical ring and arc calcification can be seen in 41% of the tumors [19]. The tumor demonstrates iso- to hypointensity on non-contrast T1-weighted and hyperintensity on T2-weighted MR images. Hypointense foci on T2-weighted images may correspond to hemorrhage or fibrous tissue. The contrast enhancement pattern is heterogeneous with a predominance at the periphery in 73% of patients (Figures 7 and 8) and homogenous in 27% [19].

### Chordoma

Chordoma is a low to intermediate, locally aggressive malignancy, presumably arising from the notochordal remnant or from benign notochordal cell tumors [20]. Skull base chordomas account for 1% of primary brain tumors and occur in the vicinity of the clivus, constituting one-third of all chordomas [21]. Chordomas can occur at all ages, with the incidence peak at 30 to 50 years and a male-to-female ratio of 2: 1 [21]. Typical clinical symptoms of clival chordomas include headache and diplopia [22]. Surgery and adjuvant radiation therapy are most frequently employed.

**Figure 9:**
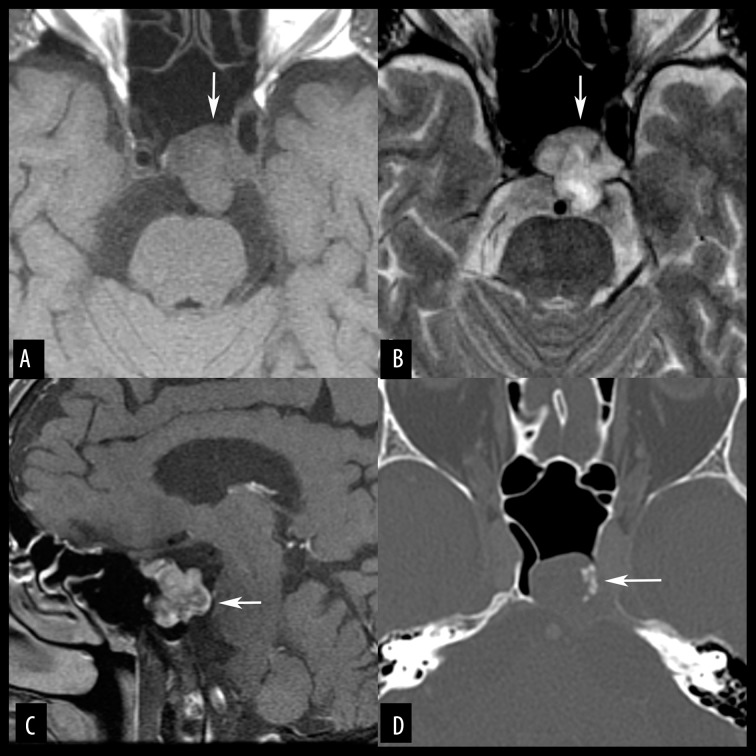
Clival chordoma in a 67-year-old man. (**A**) Axial, non-contrast, T1-weighted MR image shows a lobulated, hypointense mass lesion (arrow) in the upper part of the clivus. (**B, C**) The mass shows hyperintensity on an axial, T2-weighted MR image (**B**) and honeycomb-like enhancement on a sagittal, fat-suppressed, contrast-enhanced T1-weighted MR image (**C**). (**D**) Axial bone window CT shows cortical bone destruction of the clivus and likely a residual bone fragment within the tumor (arrow). The pathologic analysis demonstrated chordoma.

**Figure 10:**
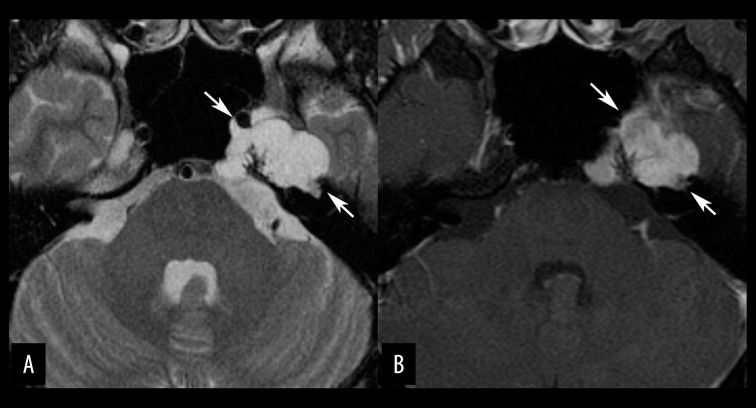
Chordoma in a 35-year-old man. (**A**) Axial, fat-suppressed, T2-weighted MR image shows a lobulated, very hyperintense mass (arrows) in the left petrous apex. The tumor extends to the left Meckel’s cave but the epicenter of the tumor lies in the left petrous bone. (**B**) Axial, contrast-enhanced MR image with fat suppression shows rather homogeneous, strong enhancement of the tumor (arrows). This is a case of lateral variants of chordoma. About one-third of skull base chordomas occur at off-midline positions.

Skull base chordoma typically forms a multilobulated expansile mass in the clivus (Figure 9). However, approximately one-third of skull base chordomas occur in off-midline positions (Figure 10) [23]. Chordomas show mild hyper- to hypodensity on non-contrast CT. Bone destruction may be more aggressive in chordomas than in chondrosarcomas (Figure 9). Chordomas show iso- to hypointensity on non-contrast T1-weighted and hyperintensity on T2-weighted MR images. Signal intensity of the tumor can be modified by intratumoral hemorrhage and calcification. Contrast-enhanced T1-weighted MR imaging shows moderate to strong enhancement with a typical honeycomb appearance [21]. The imaging characteristics of chordomas are substantially shared by chondrosarcomas, but chordomas typically arise in the midline location (clivus) and will infiltrate into surrounding tissues more often than chondrosarcomas.

### Giant cell tumor

Giant cell tumor (GCT) is a rare benign intraosseous neoplasm containing multinuclear giant cells. It is rare in the skull base and most commonly occurs in the sphenoid bone followed by the temporal bone [24]. GCT is most common in young adults, with a slight female predominance [25].

GCT shows an expansile intraosseous mass with reactive bone remodeling on CT. GCT shows low to intermediate signal intensity on both T1-weighted and T2-weighted MR images. Intra-lesional hypointensity on T2-weighted images corresponds to hemosiderin or calcification typical to GCT [24]. Contrast-enhanced T1-weighted MR imaging shows variable enhancement patterns [24].

## Middle and Posterior Skull Base

### Schwannoma

Skull base schwannomas include vestibular, trigeminal, jugular foramen and hypoglossal schwannomas. Facial nerve schwannoma commonly occurs within the temporal bone and will not be discussed in this section. Vestibular schwannoma (VS) is most common, constituting more than 80% of skull base schwannomas. Trigeminal schwannoma (TS) accounts for 1–8% of intracranial schwannomas [26], followed by jugular foramen schwannoma (JFS) at 2.9% [27]. Hypoglossal schwannoma is extremely rare. Skull base schwannomas most commonly occur between the ages of 20 to 60 years, and women are more commonly affected. VS commonly causes chronic progressive hearing loss. TS causes facial pain and paresthesia in 91% of patients [26]. JFS may cause palsies in the 9^th^ to 11^th^ cranial nerves, but may be tolerated by their opposite nerve functions [28]. As a result, up to 50% of the patients with JFS may seek medical help only after tumors have become large and hearing loss has developed [27,28]. Surgery is the treatment of choice for skull base schwannomas. Stereotactic radiosurgery or other radiotherapy techniques may be employed to follow incomplete resection.

**Figure 11:**
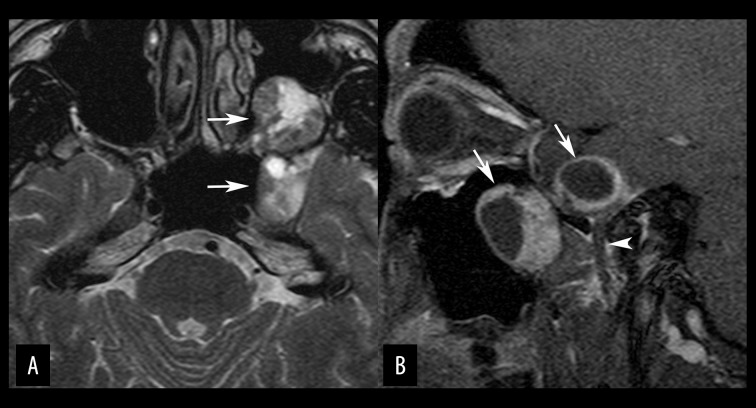
Trigeminal schwannoma in a 38-year-old man. (**A**) Axial, T2-weighted image show a dumbbell-shaped, partly cystic mass (arrows) extending from the left parasellar region to the left pterygopalatine fossa. (**B**) Sagittal, contrast-enhanced MR image with fat suppression shows both intra- and extracranial components of the tumor (arrows). The third branch of the trigeminal nerve seems normal (arrow head). Schwannoma of the second branch of the trigeminal nerve was confirmed on surgery.

**Figure 12:**
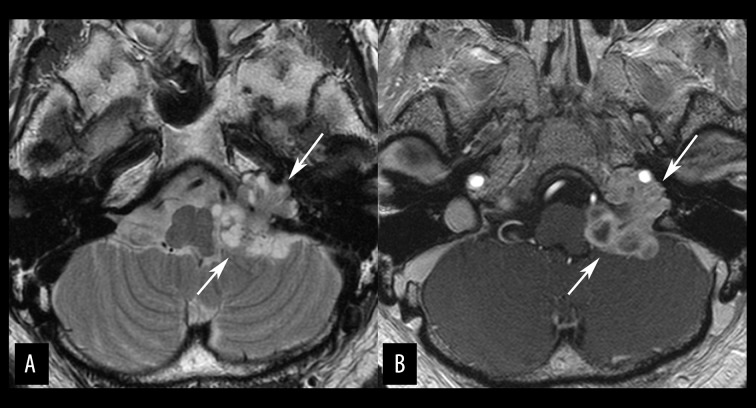
Jugular foramen schwannoma in a 66-year-old man. (**A**) Axial, T2-weighted image shows a mixed solid and cystic mass (arrows) extending through the left jugular foramen. (**B**) Axial, post contrast, T1-weighted image shows heterogeneous enhancement of the tumor (arrows).

Imaging studies show a tubular or dumbbell-shaped mass along the course of the cranial nerves with distinct margins (Figures 11, 12). Skull base schwannomas are typically hypodense on non-contrast CT, iso- to hypointense on non-contrast T1-weighted MR images and hyperintense on T2-weighted MR images. Intratumoral cyst or hemorrhage are common. Contrast-enhanced CT or MR show variable and inhomogeneous enhancement depending on the predominance of the cell-rich Antoni type A and the looser Antoni type B lesions, and the presence of cyst or hemorrhage in the tumor [29].

## Anywhere in the Skull Base

### Meningioma

Skull base meningiomas account for about 36–50% of all meningiomas [30,31]. Their common locations include the olfactory groove, the tuberculum sellae, the sphenoid ridge for the anterior skull base, petroclival and pericavernous regions for the middle skull base, and the foramen magnum and the jugular foramen for the posterior skull base. Middle-aged women are most commonly affected, which is similar to meningiomas in other locations. Meningioma is often slowly growing and asymptomatic. Larger tumors can cause both non-specific symptoms (headache and dizziness) and site-specific symptoms (anosmia, proptosis, ophthalmoplegia, and lower cranial nerve palsies). Surgery is usually employed for treatment of symptomatic meningiomas and asymptomatic tumors that are large and infiltrating. Adjuvant radiotherapy over a series of sessions or stereotaxic radiosurgery with a high single dose of radiation may be employed in cases of incomplete resection. Stereotactic radiotherapy or radiosurgery alone are indicated only for tumors that can be treated adequately with these modalities or for cases that are associated with postsurgical complications (e.g. cavernous sinus or petroclival meningiomas) [31].

**Figure 13:**
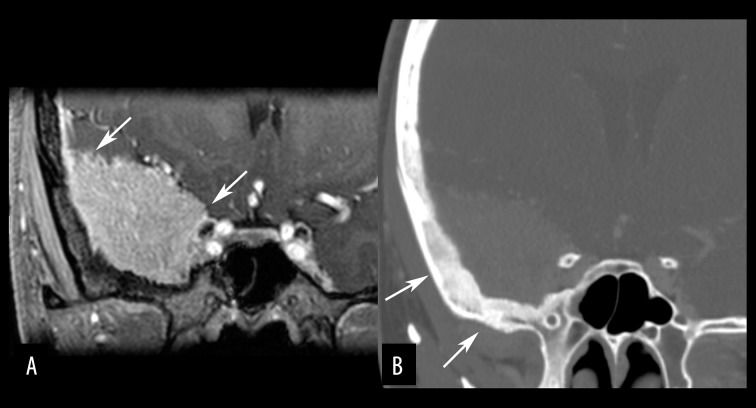
Skull base meningioma in a 29-year-old woman. (**A**) Coronal-reformatted, post contrast, T1-weighted image shows a meningioma with typical dural tail sign (arrows). (**B**) Coronal-reformatted, bone window CT image demonstrates hyperostosis of the underlying bones (arrows).

The imaging features of skull base meningiomas are similar to those of typical meningiomas, showing a sessile or lentiform, well-circumscribed mass with a broad-based dural attachment. It typically demonstrates hyperdensity on non-contrast CT, and iso- to hypointensity on both T1- and T2-weighted MR images. Uniform enhancement can be seen after contrast administration [30]. Meningiomas cause hyperostosis in the underlying bones in up to 90% of the tumors [32] (Figure 13) but can also cause osteolysis. Skull base meningioma with atypical imaging features may mimic other skull base malignancies including metastasis and multiple myeloma [33].

### Bone metastasis

It is estimated that more than one-third of people who die of cancer have bone involvement [34]. Bone metastasis often occurs in patients with breast, lung or prostate cancer, but can be found in most cases of advanced malignancies. The skull and the skull base are frequent sites for bone metastasis. Bone metastasis can be either osteolytic or osteoblastic, depending on the activities of osteoblasts, osteoclasts and relevant cytokines [34]. Common clinical symptoms include headache, nausea and local neurological deficits. Treatment of bone metastasis depends on tumor volume, lesion location, tumor pathology and the general condition of the patient. Radiotherapy and/or systemic chemotherapy are frequent choices of treatment.

Bone metastasis typically forms a destructive mass centered in the bone with no sclerotic rim on CT. Osteolytic metastasis is more common. Bone metastasis shows typically hypointense to normal bone marrow on non-contrast T1-weighted images (Figure 2). Diffusion-weighted MR images can improve detection of skull metastasis for breast and lung cancer but may be insensitive to prostate cancer [35]. Signal intensity on T2-weighted images is variable. On contrast-enhanced T1-weighted images, bone metastasis sometimes enhances to normal bone marrow signal, thus the addition of a fat suppression technique is desirable.

### Multiple myeloma

Multiple myeloma is a low grade hematologic malignancy characterized by monoclonal proliferation of B lymphocytes in the plasma cell lineage. It accounts for 10% of all hematologic tumors [36] and most commonly occurs in the elderly, with a slight male predominance [37]. Anemia and bone pain are typical symptoms found in 73% and 58% of myeloma patients, respectively [37]. Treatment depends on the disease status, ranging from observation with supportive care to chemotherapy.

Although the traditional Durie and Salmon staging system employs plain radiographs [38], CT is faster, more sensitive and better tolerated by patients [39]; although radiation exposure to the patient is a drawback [40]. Multiple myeloma typically appears as multiple, punched-out, lytic bone lesions on CT, and solitary bone plasmacytoma as a single lytic bone lesion without sclerotic borders. Skull base extramedullary plasmacytoma may erode adjacent bones.

**Figure 14:**
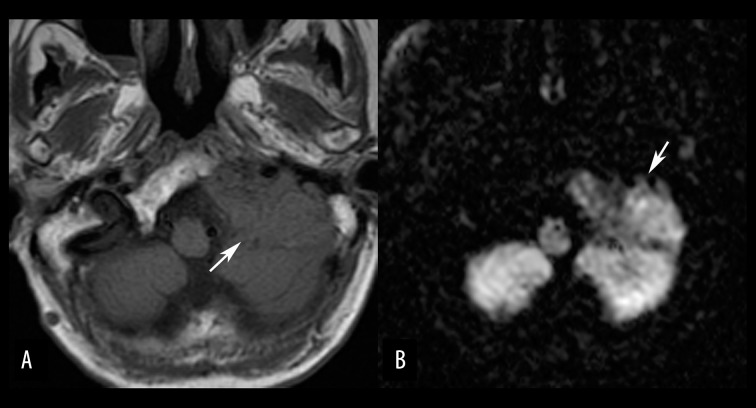
Multiple myeloma in a 76-year-old man. (**A**) Axial, non-contrast T1-weighted MR image shows a hypointense mass (arrow) destroying the left petrous bone and the left lower clivus, in contrast to normal hyperintense fatty bone marrow. (**B**) On an axial, diffusion-weighted image, the mass is hyperintense, equivalent to the brain tissue.

MRI is the most sensitive modality for detection of both diffuse bone marrow involvement and solid plasma cell tumors [39]. MR imaging shows focal myeloma lesions in approximately 30% of patients [39]. Focal myeloma lesions show hypointensity to normal fatty marrow on T1-weighted MR images and hyperintensity on T2-weighted MR images (Figure 14). Diffusion-weighted images can detect smaller myeloma lesions. Diffuse infiltration is less common and shows a diffuse decrease in the signal of the marrow on T1-weighted MR images and a variable increase in the signal of the marrow on T2-weighted MR images. Up to 30% of myeloma patients have normal-looking bone marrow signals on MR images [39]. Contrast-enhanced MR studies show marked enhancement. Multiple myeloma has multiple lesions or diffuse marrow abnormality shown on MR images. A single lesion may suggest plasmacytoma.

### Langerhans cell histiocytosis

Langerhans cell histiocytosis (LCH) is a rare histiocytic disorder characterized by single or multiple osteolytic bone lesions demonstrating histiocytic infiltration with or without invasion of the extraskeletal organs. LCH most commonly occurs in children. Bones are affected in 77% of patients with LCH [41] and therefore LCH can be a top differential diagnosis for skull base tumors in pediatric populations. Bone pain and palpable mass are typical clinical symptoms. Treatment depends on whether LCH affects a single system or many systems, and on the number and location of LCH lesions. Patients with bone involvement of the skull base are at increased risk of developing CNS involvement by LCH and endocrine abnormalities [41].

LCH appears as a soft tissue mass replacing bones of the skull base on CT. LCH shows non-specific hypointensity on non-contrast T1-weighted and mild hyperintensity on T2-weighted MR images. Uniform and strong enhancement can be observed after contrast administration [42].

## Conclusions

A variety of lesions can occur in the skull base, and both CT and MR imaging contribute beneficially to the characterization of these lesions. Skull base lesions are often most noticeable on non-contrast T1-weighted MR images. Some skull base lesions may develop in typical locations and show characteristic signals on MR images; however, variations do occur. Knowledge of clinical issues and awareness of variations of imaging features would help improve diagnostic accuracy for skull base lesions.
